# Comparison of the Efficacy and Safety of Different ACE Inhibitors in Patients With Chronic Heart Failure: A PRISMA-Compliant Network Meta-Analysis

**DOI:** 10.1097/MD.0000000000002554

**Published:** 2016-02-12

**Authors:** WeiPing Sun, HaiBin Zhang, JinCheng Guo, XueKun Zhang, LiXin Zhang, ChunLei Li, Ling Zhang

**Affiliations:** From the Department of Cardiology, Beijing Luhe Hospital, Capital Medical University (WS, HZ, JG, XZ, LZ, CL); and Department of Epidemiology and Health Statistics, School of Public Health, Capital Medical University (LZ), Beijing, China.

## Abstract

Heart failure is a public health problem and a great economic burden for patients and healthcare systems. Suppression of the renin–angiotensin system (RAS) by angiotensin-converting enzyme (ACE)-inhibitors remains the mainstay of treatment for heart failure. However, the abundance of ACE inhibitors makes it difficult for doctors to choose.

We performed this network meta-analysis of ACEIs in patients with heart failure in order to address this area of uncertainty.

We searched PubMed, Embase, CENTRAL, and Medline.

Any randomized controlled trial evaluating the efficacy and safety of captopril, enalapril, lisinopril, ramipril, or trandolapril or combined interventions of 2 or more of these drugs.

Two reviewers extracted the data and made the quality assessment. At first, we used Stata software (version 12.0, StataCorp, College Station, TX) to make traditional pairwise meta-analyses for studies that directly compared different interventions. Then, network meta-analysis was performed using WinBUGS (version 1.4.3, MRC Biostatistics Unit, Cambridge, UK).

A total of 29 studies were included. Lisinopril was associated with a higher rate of all-cause mortality compared with placebo (odds ratio 65.9, 95% credible interval 1.91 to 239.6) or ramipril (14.65, 1.23 to 49.5). Enalapril significantly reduced systolic blood pressure when compared with placebo (standardized mean differences −0.6, 95% credible interval −1.03 to −0.18). Both captopril (odds ratio 76.2, 95% credible interval 1.56 to 149.3) and enalapril (274.4, 2.4 to 512.9) were associated with a higher incidence of cough compared to placebo.

Some important outcomes such as rehospitalization and cardiac death were not included. The sample size and the number of studies were limited, especially for ramipril.

Our results suggest that enalapril might be the best option when considering factors such as increased ejection fraction, stroke volume, and decreased mean arterial pressure. However, enalapril was associated with the highest incidence of cough, gastrointestinal discomfort, and greater deterioration in renal function. Trandolapril ranked first in reducing systolic and diastolic blood pressure. Ramipril was associated with the lowest incidence of all-cause mortality. Lisinopril was the least effective in lowering systolic and diastolic blood pressure and was associated with the highest incidence of all-cause mortality.

## Abstract 2

Supplemental Digital Content is available in the text

## INTRODUCTION

Heart failure (HF) is a public health problem leading to a great economic burden for both individual patients and healthcare systems. Approximately 1% to 2% of the adult population in developed countries suffers HF, with the prevalence rising to ≥10% among persons 70 years of age or older.^1,2^ In the United States, between 20% and 27% of patients hospitalized with heart failure are readmitted within 30 days of discharge.^3^ Heart failure costs 1% to 2% of healthcare resources, due to repeated hospitalization and extended inpatient days.^1^

Inhibition of the renin–angiotensin system (RAS) via angiotensin-converting enzyme (ACE)-inhibitors is the main treatment for heart failure. Because ACE inhibitors have a modest effect on the remodeling of left ventricular (LV) to some extent, the European Society of Cardiology (ESC) Guidelines for HF recommend that ACE inhibitors be prescribed immediately after HF is diagnosed.^4^ Two randomized controlled trials have demonstrated that ACE inhibitors therapy decreased mortality.^5,6^ These findings are similar with the results from a meta-analysis including short-term (3 months), placebo-controlled randomized controlled trials.^7^

However, there are so many ACE inhibitors that doctors are uncertain, which is the most effective and should be chosen first. To date, there is no meta-analysis comparing the efficacy of different ACE inhibitors in patients with heart failure. Therefore, we performed this network meta-analysis of ACEI in patients with heart failure in order to address this area of uncertainty.

## METHODS

### Eligibility Criteria

1. Participants: inclusion criterion—patients with chronic heart failure (New York Heart Association [NYHA] class II or III); exclusion criteria—patients with chronic kidney disease (CKD) or acute myocardial infarction (AMI).
2. Interventions and comparisons: inclusion criteria—any randomized controlled trial (RCT) evaluating the efficacy and safety of either captopril, enalapril, lisinopril, ramipril, or trandolapril or combined interventions of 2 or more interventions.
3. Types of study: inclusion criteria—randomized controlled trials (RCTs); exclusion criteria—quasi RCTs, cohort studies, case-control studies, case series, case reports, reviews, meta-analyses, animal studies, comments, and letters.
4. Language: no restriction. However, we excluded studies if languages other than English or Chinese could not be adequately translated through Google translate.

### Search Method and Study Selection

The following databases were searched: Embase (from 1974 to Nov 2014), PubMed (from 1966 to Nov 2014), the Cochrane Central Register of Controlled Trials (CENTRAL) (the Cochrane Library, most recent issue), and Medline (from 1966 to Nov 2014). A complete search strategy is listed in Supplemental File 1. In addition, we searched the references of included studies and reviews or meta-analyses with a similar topic to minimize the possibility of omitted studies.

Two authors independently selected the studies after reading the title and abstract. Any disagreement between 2 authors was resolved by discussion. If there was no consensus, a third reviewer was consulted.

Ethical approval was not necessary because no primary patients’ data were included.

### Data Extraction and Quality Assessment

Two authors extracted first author, publication year, comparison, sample size, country, setting (single center or multicenter), proportion of men, age, maximum follow-up duration from included studies. We used odds ratios (ORs) with 95% confidence interval (CI) for direct evidences or 95% credible intervals (CrI) for indirect evidences to report dichotomous data. For continuous variable (eg, ejection fraction, stroke volume, and blood pressure), we applied standardized mean differences (SMDs) with 95% CI for direct evidences or CrI for indirect evidences.

For missing data, we carried out an intention-to-treat analysis if possible.^8^ For dichotomous data, when the included studies used a perprotocol analysis, we used the data that the studies supplied. We then conducted sensitivity analyses through best-worst scenarios (good outcome in the active group and bad outcome in the control or another active group) and worst-best scenarios (contrary to the previous). For continuous data, we only performed the perprotocol analysis.

Cochrane risk of bias tool was put into use to evaluate the risk of bias.^9^ There were 7 domains in the tool: random sequence generation, allocation concealment, blinding of participants and personnel, blinding of outcome assessment, incomplete outcome data, selective reporting, and other bias (if there was commercial funding, early discontinuation of the study or baseline imbalance, we judged the domain as high risk of bias). Two authors independently made judgments about each domain (low risk of bias, high risk of bias, or unclear risk of bias).

### Statistical Analysis

To begin with, we used Stata software (version 12.0, StataCorp, College Station, TX) to make pairwise meta-analyses. DerSimonian and Laird random effects model was applied.^10^ We used the chi-square test and *I*-square test to test heterogeneity.^11^*I*^2^ exceeded 50% was considered as high heterogeneity and *I*^2^ under 25% was considered as no heterogeneity.^9^ For publication bias, we used a funnel plot if the number of included studies in 1 pair of the comparison was >10.^12^ Further, we used a linear regression method proposed by Egger et al to quantify the funnel asymmetry.^13^ We evaluate the loop inconsistency between direct and indirect results through ifplot command. Then, we used WinBUGS (version 1.4.3, MRC Biostatistics Unit, Cambridge, UK) with a random effects model proposed by Chaimani to perform network meta-analyses. We applied the hierarchical Bayesian model with noninformative priors.^14^ In the network meta-analysis, the posterior parameters were calculated by Markov chain Monte Carlo methods.^15^ After an initial burn-in of 50,000, we operated another 100,000 iterations.^14^ In order to make the rank of treatments, we used the surface under the cumulative ranking probabilities (SUCRA).^16^ To test the stability of the results, we performed sensitivity analyses by excluding studies with a high risk of bias. Considering that age might influence the results, we made a meta-regression for age. We chose the mean age as the covariate. When the study only supplied a range of ages, we calculated the mean age by dividing equally the sum of the upper and lower limits; when the study supplied sex-specific ages, we calculated the mean age by using the following formula: (men's mean age ×the number of men +women's mean age ×the number of women)/(the total number of patients). At last, the robustness of the model was detected with R (version 3.1.1, R Foundation for Statistical Computing, Vienna, Austria) through computing the posterior mean residual deviance. The model fitted the data well if posterior mean residual deviance approximated data points.^15^

## RESULTS

### Study Selection

**FIGURE 1:**
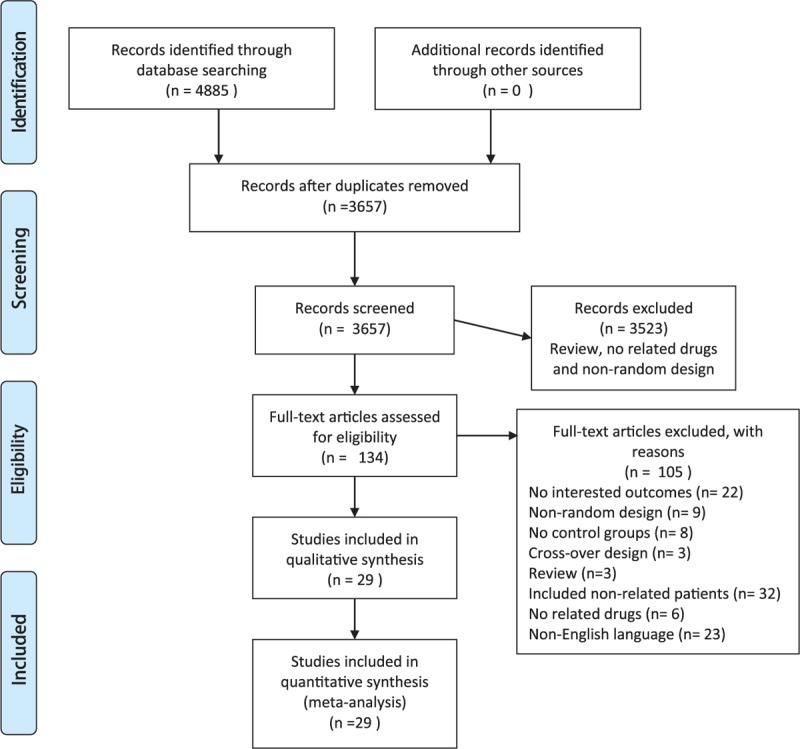
The PRISMA flow diagram.

Figure 1 shows the PRISMA flow diagram. We performed search on Nov 27th, 2014, and found 4885 references. After taking away of 1228 duplicate articles, we screened 3657 records through their abstracts and titles. A total of 134 publications were eligible for full-text screening; however, others were not selected for different reasons (eg, review, no related drugs, and nonrandom design). Finally, 29 studies were included in the meta-analysis.^17–45^ Totally, 104 studies were excluded: 32 studies included nonrelated patients, in 23 studies the language was not English, 22 studies reported no outcomes of interest, 9 study designs were nonrandom, 8 studies were without a control group, 6 studies were without related drugs, 3 study designs were crossover, and 3 studies were reviews.

### Characteristics of Included Studies

Table 1 provides a summary of the included studies. A total of 2099 participants were included in this meta-analysis. Webster 1985 d and Webster 1985 A were 2 publications of the same study. Only 1 study included ramipril. The study sample size ranged from 16 to 287. These studies were published between 1985 and 2010. Most studies were single-center studies. None of the studies were performed in Africa. Most of the included studies (26/29, 89.6%) did not specify the population type. Two studies mentioned outpatients, and 1 study mentioned ambulatory patients. Most of the included studies (19/29, 65.5%) only mentioned chronic heart failure. Nine studies referred to chronic heart failure without preserved ejection fraction (4 studies with an ejection fraction < 45%, 4 with an ejection fraction < 40%, and 1 with an ejection fraction < 30%). One study included patients with preserved ejection fraction.

**TABLE 1:**
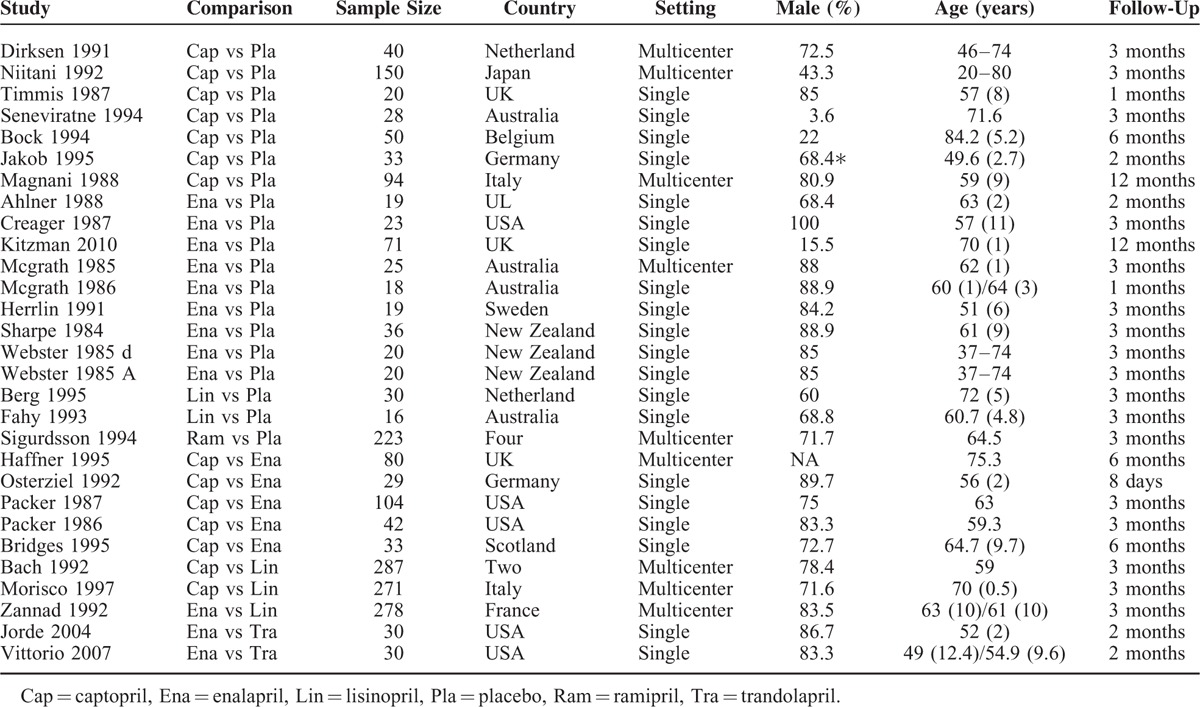
Characteristics of Included Studies

### Risk of Bias in the Included Studies

**FIGURE 2:**
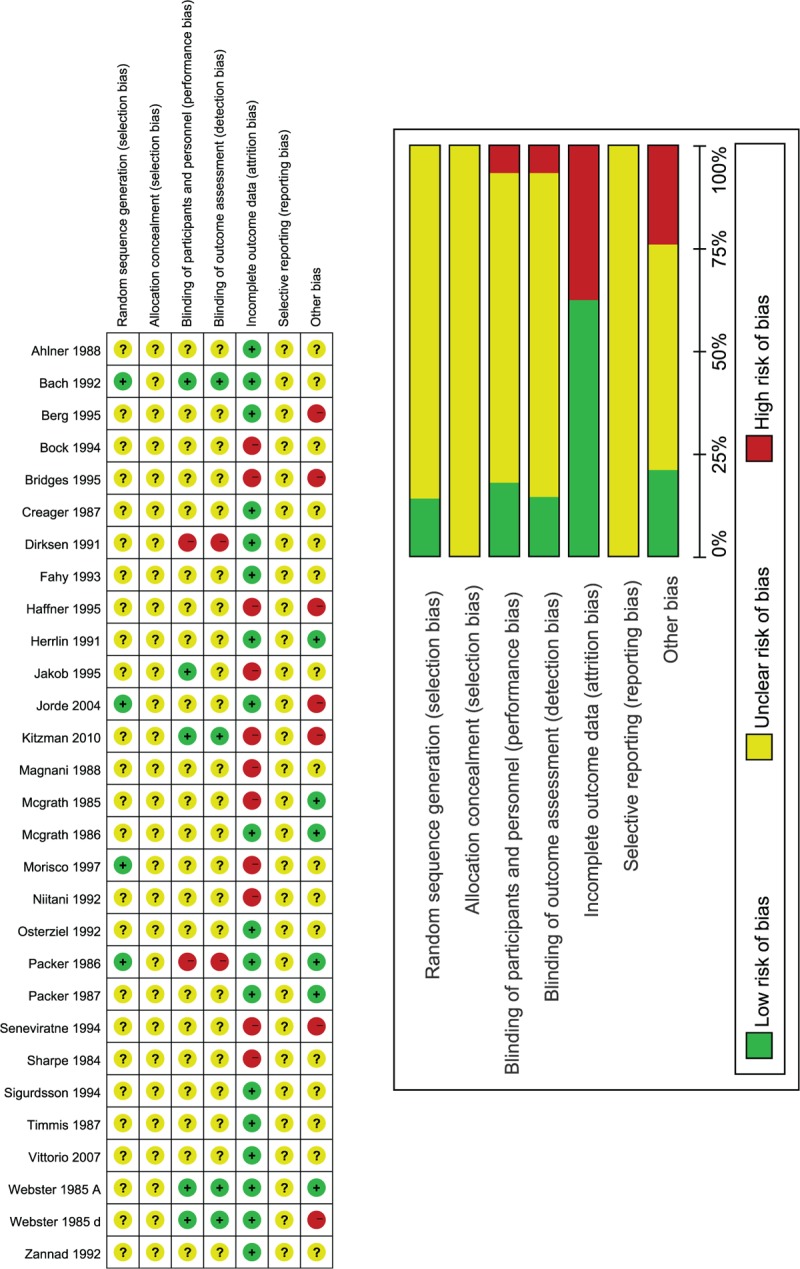
Risk of bias of all included studies.

The risk of bias is indicated in Figure 2. For random sequence generation domain, 4 studies were rated as low risk of bias. None described adequate allocation concealment. In terms of blinding of participants and personnel, 5 studies had a low risk of bias. Two studies did not blind to participants and personnel. Considering blinding of the outcome assessment domain, 4 studies were considered as a low risk of bias. Two studies had a high risk of bias in blinding of the outcome assessment. Eighteen studies had a low risk of bias regarding incomplete outcome data domain; in contrast, 11 studies were considered as a high risk of bias. All studies had an unclear risk of bias with regard to selectively in reporting results. Seven studies had a high risk of bias in other biases domain (eg, funding, study early discontinuation and baseline imbalance).

### Primary Outcomes

#### All-Cause Mortality

All-cause mortality included pulmonary edema, ventricular fibrillation, acute myocardial infarction, complications after surgical intervention for colon cancer during the study, sudden death (reason unknown), stroke, progressive renal insufficiency, and severe heart failure.

**FIGURE 3:**
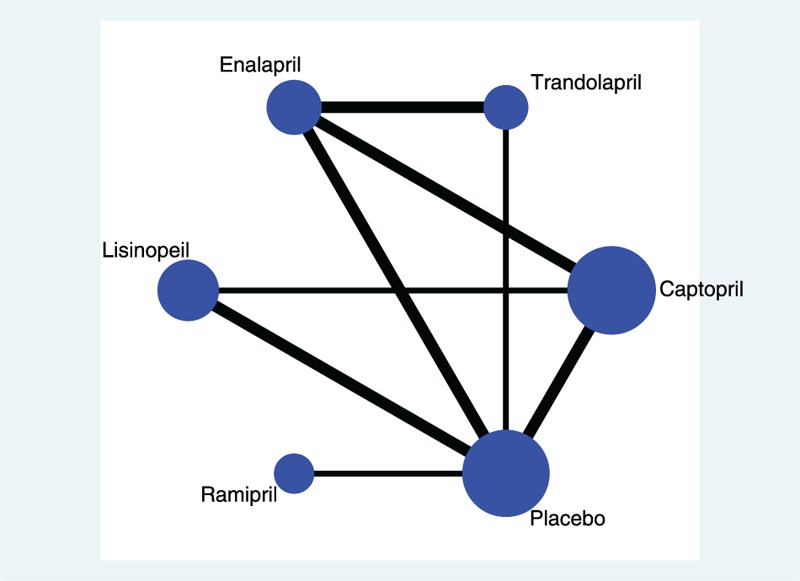
Network plot of treatment comparisons. The size of the nodes represents the total sample size of treatments. The lines’ thickness corresponds to the number of trials that compare each other. Cap = captopril, Ena = enalapril, Lin = lisinopril, Pla = placebo, Ram = ramipril, Tra = trandolapril.

The network of comparisons is indicated in Figure 3. Thirteen studies (1022 patients) with 4 drugs (captopril, enalapril, lisinopril, and ramipril) and placebo were included. Lisinopril was associated with higher all-cause mortality compared with placebo (OR 65.9, 95%CrI 1.91 to 239.6) or ramipril (14.65, 1.23 to 49.5). No significant differences were found in the other comparisons. Details of the comparisons are shown in Supplemental Table 1.

#### Stroke Volume

Six studies (423 patients) with 3 drugs (captopril, enalapril, and lisinopril) and placebo were included. No significant differences were found among the 4 interventions. Details of the comparisons are shown in Supplemental Table 2.

#### Ejection Fraction

Five studies (453 patients) with 3 drugs (captopril, enalapril, and lisinopril) and placebo were included. No significant differences were found among the 4 interventions. Details of the comparisons are shown in Supplemental Table 3.

### Secondary Outcomes

#### Blood Pressure

Blood pressure outcomes were reported as systolic blood pressure (SBP), diastolic blood pressure (DBP), and mean arterial pressure (MAP). For SBP, 9 studies (606 patients) with 4 drugs (captopril, enalapril, lisinopril, and trandolapril) and placebo were included. Enalapril significantly reduced SBP compared to placebo (SMD −0.6, 95%CrI −1.03 to −0.18). For DBP, 7 studies (563 patients) with 4 drugs (captopril, enalapril, lisinopril, and trandolapril) and placebo were included in the meta-analysis. No significant differences were found among the 5 interventions. For MAP, 9 studies (427 patients) with 2 drugs and placebo were included. No significant differences were found among the 3 interventions. Details of the comparisons are shown in Supplemental Tables 4–6.

#### Cough

Four studies (341 patients) with 2 drugs (captopril and enalapril) and placebo were included in the meta-analysis. Both captopril (OR 76.2, 95%CrI 1.56–149.3) and enalapril (274.4, 2.4–512.9) were associated with a higher incidence of cough compared to placebo. There was no significant difference in cough between captopril and enalapril (0.64, 0.1–1.78). Details of the comparisons are shown in Supplemental Table 7.

#### Deterioration of Renal Function

Four studies (713 patients) with 3 drugs (captopril, enalapril, and lisinopril) and placebo were included in the meta-analysis. Captopril was associated with a lower incidence of renal function deterioration compared with enalapril (OR 0.04, 95%CrI 0.002–0.14). No significant differences were found in the other comparisons. Details of the comparisons are shown in Supplemental Table 8.

#### Gastrointestinal Discomfort

Six studies (777 patients) with 3 drugs (captopril, enalapril, and lisinopril) and placebo were included in the meta-analysis. No significant differences were found among the 4 interventions. Details of the comparisons are shown in Supplemental Table 9.

#### Comparisons Between Pairwise and Network Meta-Analyses

The results of the pairwise and network meta-analyses are shown in Supplemental Tables 1–9. The CI from the pairwise meta-analyses and the CrI from the network meta-analyses nearly overlapped, indicating that there were no inconsistencies between the direct and mixed comparisons.

#### Ranking of Treatments

**FIGURE 4:**
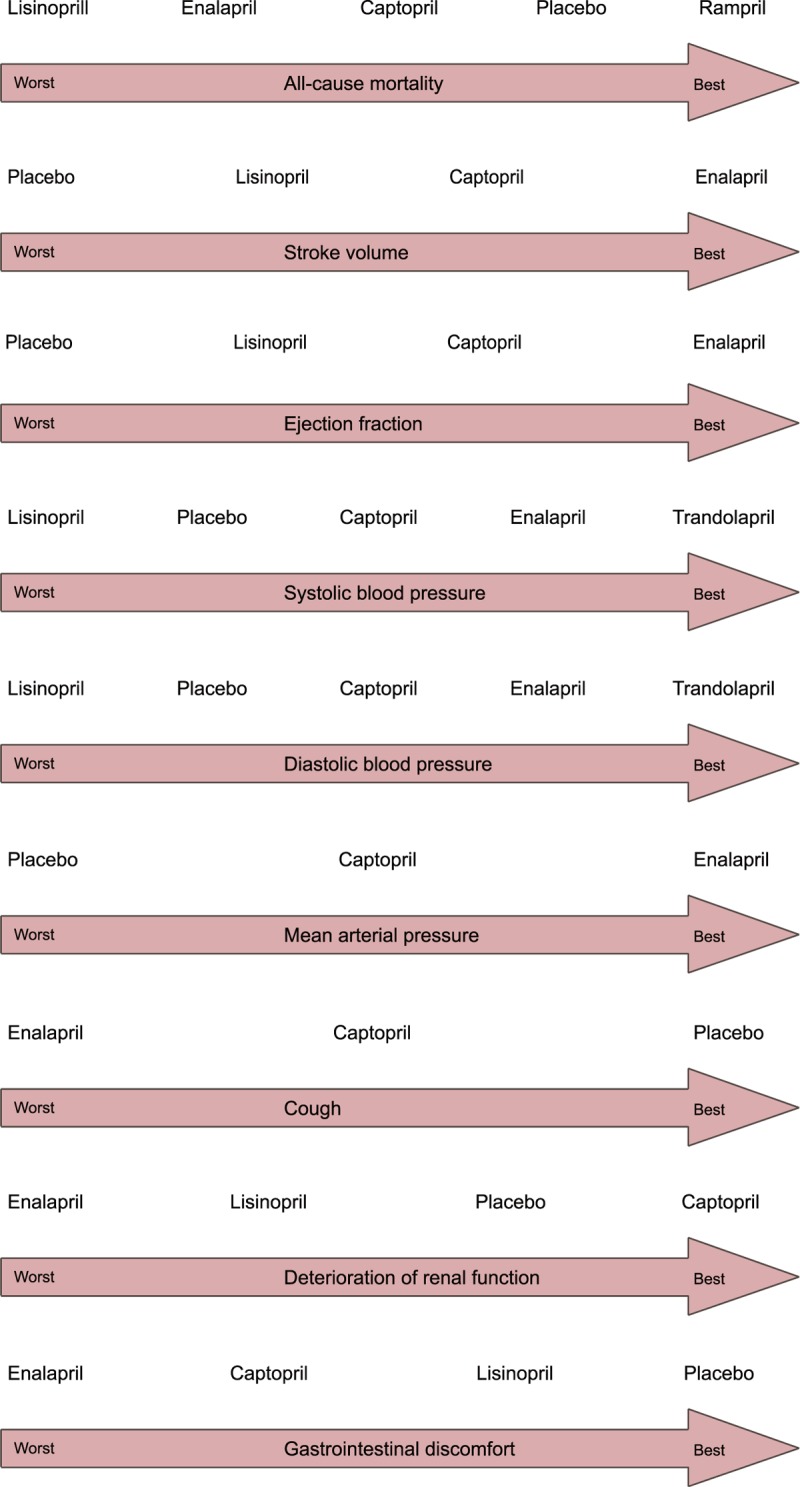
Ranking of treatments. Cap = captopril, Ena = enalapril, Lin = lisinopril, Pla = placebo, Ram = ramipril, Tra = trandolapril.

In Figure 4, we summarize the ranking of all the interventions in terms of all outcomes. For all-cause mortality, ramipril was associated with the lowest mortality and lisinopril with the highest. For increasing ejection fraction and stroke volume, enalapril was the most effective and the placebo ranked the lowest in efficacy. For reducing SBP and DBP, trandolapril ranked first and lisinopril ranked last. For decreasing MAP, enalapril was the most effective, whereas the placebo was the least effective. The placebo was associated with the lowest incidence of cough and enalapril with the highest. Captopril was associated with the lowest the incidence of renal function deterioration, whereas enalapril was associated with the highest. The placebo had the lowest incidence of gastrointestinal discomfort and enalapril the highest.

#### Publication Bias, Sensitivity Analyses, and Meta-Regression

Funnel plots were not performed because the number of included studies in 1 comparison was <10. Overall, sensitivity analyses showed that the results were stable. The result of a meta-regression indicated that age was not associated with differences in the outcome of different drug treatments (*P* > 0.05).

## DISCUSSION

### Summary of Evidence

For primary outcomes (all-cause mortality, stroke volume, and ejection fraction), only lisinopril was associated with a higher incidence of all-cause mortality compared with placebo or ramipril. For secondary outcomes (blood pressure, cough, deterioration of renal function, and gastrointestinal discomfort), enalapril significantly reduced systolic blood pressure compared with placebo, whereas both captopril and enalapril were associated with a higher incidence of cough compared to placebo. No significant differences were found among the other comparisons of all the outcomes.

### Comparison With Other Studies

This is the first network meta-analysis to compare the efficacy and safety of ACE inhibitors in patients with chronic heart failure (NYHA II or III). For blood pressure control and safety outcomes (all-cause mortality, cough, deterioration of renal function, and gastrointestinal discomfort), a previous meta-analysis^46^ focused on patients with hypertension and showed similar results for all of the outcomes except for all-cause mortality. For all-cause mortality, results from the network meta-analysis were positive, whereas results from the direct meta-analysis were negative or absent. Relevant studies were limited, and the sample size was small; therefore, the discrepancies between direct evidence and network evidence might be due to random error. The 2012 guidelines for management of heart failure recommend that ACE inhibitors should be used in all patients with an EF ≤ 40% to reduce the risk of HF hospitalization and premature death (CLASS IA).^4^ However, the guidelines do not mention all-cause mortality. This might, in part, be because so many factors in clinical settings can result in the death of a patient, and it is difficult to make a direct correlation between treatment with ACEI and a reduction in all-cause mortality.

### Limitations

First, some important outcomes such as re-hospitalization and cardiac death were not included in this analysis because the relevant data were not supplied. Second, most studies were single-center studies, few were performed in Asia and none were performed in Africa; therefore, the results should be generalized with caution. Another limitation is the small sample size and the limited number of studies, especially for ramipril (only 1 study). Moreover, although we included 5 ACE inhibitors, some outcomes did not include all 5, which made the results less comprehensive. In addition, the duration of follow-up differed among the studies and the data were insufficient to perform subgroup analyses, which could lead to imprecise determination of time-related outcomes. Last, our article used summary data rather than individual patient data, which could introduce some biases at the individual patient level.

## CONCLUSION

When considering factors such as increased ejection fraction, stroke volume, and decreasing mean arterial pressure, our results suggest that enalapril was the most effective ACE inhibitor. However, enalapril was also associated with the highest incidence of cough, as well as renal function deterioration and gastrointestinal discomfort. An increase in all-cause mortality combined with a limited effect on reducing systolic and diastolic blood pressure made lisinopril the worest choice among the ACE inhibitors evaluated. Ramipril was associated with the lowest incidence of all-cause mortality. Trandolapril ranked first in reducing systolic and diastolic blood pressure. More high quality, randomized controlled trials of longer duration and with a larger sample size should be performed to confirm these results and explore the impact of different ACE inhibitors on other important outcomes such as rehospitalization and cardiac death.

## Supplementary Material

Supplemental Digital Content
