# Supplementary material for: Comparison of the Efficacy and Safety of Different ACE Inhibitors in Patients With Chronic Heart Failure: A PRISMA-Compliant Network Meta-Analysis
Source: Medicine (Baltimore). 2016 Feb 12;95(6):e2554. doi: 10.1097/MD.0000000000002554 (PMC4753869; doi:10.1097/MD.0000000000002554)
Supplement: Supplemental Digital Content [file medi-95-e2554-s001.doc]

Supplemental File 1. Search strategy.

***A. Cochrane Central Register of Controlled Trials (CENTRAL) [in the Cochrane Library]***

#1 MeSH descriptor: [Heart Failure] explode all trees

#2 (((Heart or Cardiac or Cardial or Myocardial or Coronary) near/2 (Failure* or Decompensation* or Incompetence* or Insufficien* or "stand still" or standstill)) or Cardio Renal Syndrome* or Cardiorenal Syndrome* or Cardio-Renal Syndrome* or Reno cardiac Syndrome* or Renocardiac Syndrome* or Reno-cardiac Syndrome* or Paroxysmal Dyspnea* or Cardiac Asthma or Cardiac Edema* or heart edema* or ((cardiogenic or cardiac or cardiovascular or heart) near/1 shock*) or ((cardiopulmonary or cardiorespiratory) near/1 (insufficiency or arrest* or collaps* or failure*)) or CHF or left ventricular diastolic dysfunction or forward failure* or low cardiac output* or low heart output* or low output syndrome* or ((cardiac or circulation or circulatory or heart or cardiopulmonary) near/1 arrest*) or asystole or asystolia or asystoly or heart arrhythmia* or cardiac death* or heart death* or heart outflow tract obstruction* or ventricular outflow tract obstruction* or ventricular outflow obstruction* or ventricle obstruction* or heart outflow obstruction* or heart obstruction* or ventric* failure* or ventric* insufficien* or ventric* dysfunction* or systolic dysfunction* or diastolic overload* or systolic overload* or heart overload* or ventric* overload* or ventric* tension overload* or ventric* pressure overload* or ventric* volume overload* or ventric* strain* or high cardiac output* or high output failure* or propofol infusion syndrome* or propofol related infusion syndrome*):ti,ab,kw

#3 #1 or #2

#4 MeSH descriptor: [Fosinopril] explode all trees

#5 MeSH descriptor: [Lisinopril] explode all trees

#6 MeSH descriptor: [Perindopril] explode all trees

#7 MeSH descriptor: [Enalapril] explode all trees

#8 MeSH descriptor: [Captopril] explode all trees

#9 (Accupril or Accuprin or Accupro or Accupron or Ace-bloc or Acenorm or Acenor-m or Aceon or Acepress or Acepril or Aceprilex or Acequin or Acerbon or Aceril or Aceten or Acuitel or Acuprel or Acupril or Adocor or Alapril or Alfaken or Alopresin or Altran or Amprace or Apuzin or Asig or Asisten or Benazapril or Benazepril or Benzazepril or Bpnorm or Briem or Capace or Capocard or Caposan or Capoten or Capotena or Capotril or Capril or Captace or Captensin or Capti or Captoflux or Captohexal or Captolane or Captomax or Capton or Captopren or Captopril or Captoprilan or Captoril or Captral or Carace or Cardiopril or Cardipril or Catona or Catoplin or Catopril or Cesplon or "CGS-14824A" or "CGS-14824-A" or "CI 906" or "CI906" or "CI-906" or Cibacen or Cibacene or Cipril or Conan or Coric or Coversum or Coversyl or Cryopril or Dapril or Debax or Dexacap or Dynacil or Ecapres or Ecaten or Ednyt or Enalapril* or Epicordin or Epsitron or Farcopril or Farmoten or Fibsol or Fosenopril or Fosinil or Fosinonorm or Fosinopril or Fosinorm or Fosipres or Fositen or Fositens or Fovas or Fozitec or Hiperil or Hiperlex or Hypopress or Hypotensor or "Ici 209000" or "Ici pharma 209000" or "Ici209000" or Inopril or Insucar or Iopril or Isopresol or Katopil or Ketanine or Keyerpril or Korec or Labopal or Lapril or Linopril or Linvas or Lipril or "Lisi abz" or Lisibeta or Lisigamma or Lisihexal or Lisinopril or Lisipril or Lisodur or Lisopress or Lisopril or Lisoril or Lispril or Listril or Locap or Lopirin or Lopril or Lotensin or Lysinopril or Medepres or Midrat or Minitent or "Mk 0521" or "MK 421" or "MK 422" or "Mk 521" or "Mk 522" or "Mk0521" or "MK421" or "MK-421" or "MK422" or "MK-422" or "Mk521" or "MK-521" or "Mk522" or Monopril or Naprilene or Newace or Nolectin or Noperten or Novatec or "Oltens ge" or "Pd 109452 2" or "Pd 1094522" or "PD 109452-2" or "PD-109,452-2" or "Pd109452 2" or "Pd1094522" or Perindopril or Perstarium or Petacilon or Pirindopril or Praten or "Pres iv" or Presiten or Prestarium or Primace or Prinil or Prinivil or Quinalapril or Quinapril or Quinaten or Quinazil or Quinipril or Renitec or Renitek or Rilcapton or Ropril or "S 9490" or "S 9490 3" or "S 94903" or "S 9490-3" or "S9490" or "S-9490" or Sapril or Sinopril or Smarten or "SQ 14,225" or "SQ 14,534" or "SQ 14225" or "SQ 14534" or "Sq 28 555" or "SQ 28,555" or "Sq 28555" or "SQ 28555" or "SQ14,225" or "SQ-14,225" or "SQ14,534" or "SQ-14,534" or "SQ14225" or "SQ-14225" or "SQ14534" or "SQ-14534" or "SQ28,555" or "SQ-28,555" or "Sq28.555" or "SQ28555" or "SQ-28555" or Staril or Tenofax or Tensicap or Tensiomen or Tensiomin or "Tenso Stop" or Tensobon or Tensocardil or Tensoprel or Tensopril or Tensoril or Tensyn or Tenzib or Topace or Toprilem or Typril-ace or Vasopril or Vasosta or Vasotec or Vivatec or Xanef or Zapto or Zestomax or Zestril or Zorkaptil):ti,ab,kw

#10 #4 or #5 or #6 or #7 or #8 or #9

#11 #3 and #10 in Trials

***B. EMBASE via Ovid SP***

1. exp Heart Failure/ or (((Heart or Cardiac or Cardial or Myocardial or Coronary) adj2 (Failure? Or Decompensation? or Incompetence? Or Insufficien$ or "stand still" or standstill)) or Cardio Renal Syndrome? or Cardiorenal Syndrome? or Cardio-Renal Syndrome? or Reno cardiac Syndrome? or Renocardiac Syndrome? or Reno-cardiac Syndrome? or Paroxysmal Dyspnea? or Cardiac Asthma or Cardiac Edema? Or heart edema? Or ((cardiogenic or cardiac or cardiovascular or heart) adj shock$) or ((cardiopulmonary or cardiorespiratory) adj (insufficiency or arrest$ or collaps$ or failure?)) or CHF or left ventricular diastolic dysfunction or forward failure? Or low cardiac output? Or low heart output? Or low output syndrome? or ((cardiac or circulation or circulatory or heart or cardiopulmonary) adj arrest?) or asystole or asystolia or asystoly or heart arrhythmia? Or cardiac death? or heart death? or heart outflow tract obstruction? or ventricular outflow tract obstruction? or ventricular outflow obstruction? or ventricle obstruction? Or heart outflow obstruction? Or heart obstruction? Or ventric$ failure? Or ventric$ insufficien$ or ventric$ dysfunction? Or systolic dysfunction? Or diastolic overload? or systolic overload? or heart overload? or ventric$ overload? Or ventric$ tension overload? Or ventric$ pressure overload? Or ventric$ volume overload? Or ventric$ strain? Or high cardiac output? or high output failure? or propofol infusion syndrome? or propofol related infusion syndrome?).mp.
2. Fosinopril/ or Lisinopril/ or Perindopril/ or Enalapril/ or Captopril/ or Quinapril/ or (Accupril or Accuprin or Accupro or Accupron or Ace-bloc or Acenorm or Acenor-m or Aceon or Acepress or Acepril or Aceprilex or Acequin or Acerbon or Aceril or Aceten or Acuitel or Acuprel or Acupril or Adocor or Alapril or Alfaken or Alopresin or Altran or Amprace or Apuzin or Asig or Asisten or Benazapril or Benazepril or Benzazepril or Bpnorm or Briem or Capace or Capocard or Caposan or Capoten or Capotena or Capotril or Capril or Captace or Captensin or Capti or Captoflux or Captohexal or Captolane or Captomax or Capton or Captopren or Captopril or Captoprilan or Captoril or Captral or Carace or Cardiopril or Cardipril or Catona or Catoplin or Catopril or Cesplon or "CGS-14824A" or "CGS-14824-A" or "CI 906" or "CI906" or "CI-906" or Cibacen or Cibacene or Cipril or Conan or Coric or Coversum or Coversyl or Cryopril or Dapril or Debax or Dexacap or Dynacil or Ecapres or Ecaten or Ednyt or Enalapril$ or Epicordin or Epsitron or Farcopril or Farmoten or Fibsol or Fosenopril or Fosinil or Fosinonorm or Fosinopril or Fosinorm or Fosipres or Fositen or Fositens or Fovas or Fozitec or Hiperil or Hiperlex or Hypopress or Hypotensor or "Ici 209000" or "Ici pharma 209000" or "Ici209000" or Inopril or Insucar or Iopril or Isopresol or Katopil or Ketanine or Keyerpril or Korec or Labopal or Lapril or Linopril or Linvas or Lipril or "Lisi abz" or Lisibeta or Lisigamma or Lisihexal or Lisinopril or Lisipril or Lisodur or Lisopress or Lisopril or Lisoril or Lispril or Listril or Locap or Lopirin or Lopril or Lotensin or Lysinopril or Medepres or Midrat or Minitent or "Mk 0521" or "MK 421" or "MK 422" or "Mk 521" or "Mk 522" or "Mk0521" or "MK421" or "MK-421" or "MK422" or "MK-422" or "Mk521" or "MK-521" or "Mk522" or Monopril or Naprilene or Newace or Nolectin or Noperten or Novatec or "Oltens ge" or "Pd 109452 2" or "Pd 1094522" or "PD 109452-2" or "PD-109,452-2" or "Pd109452 2" or "Pd1094522" or Perindopril or Perstarium or Petacilon or Pirindopril or Praten or "Pres iv" or Presiten or Prestarium or Primace or Prinil or Prinivil or Quinalapril or Quinapril or Quinaten or Quinazil or Quinipril or Renitec or Renitek or Rilcapton or Ropril or "S 9490" or "S 9490 3" or "S 94903" or "S 9490-3" or "S9490" or "S-9490" or Sapril or Sinopril or Smarten or "SQ 14,225" or "SQ 14,534" or "SQ 14225" or "SQ 14534" or "Sq 28 555" or "SQ 28,555" or "Sq 28555" or "SQ 28555" or "SQ14,225" or "SQ-14,225" or "SQ14,534" or "SQ-14,534" or "SQ14225" or "SQ-14225" or "SQ14534" or "SQ-14534" or "SQ28,555" or "SQ-28,555" or "Sq28.555" or "SQ28555" or "SQ-28555" or Staril or Tenofax or Tensicap or Tensiomen or Tensiomin or "Tenso Stop" or Tensobon or Tensocardil or Tensoprel or Tensopril or Tensoril or Tensyn or Tenzib or Topace or Toprilem or Typril-ace or Vasopril or Vasosta or Vasotec or Vivatec or Xanef or Zapto or Zestomax or Zestril or Zorkaptil).mp.
3. 1 and 2
4. exp animals/ or exp invertebrate/ or animal experiment/ or animal model/ or animal tissue/ or animal cell/ or nonhuman/
5. human/ or normal human/ or human cell/
6. 4 and 5
7. 4 not 6
8. 3 not 7
9. (random$ or factorial$ or crossover$ or (cross over$) or cross-over$ or placebo$ or (doubl$ adj blind$) or (singl$ adj blind$) or assign$ or allocat$ or volunteer$).mp. or crossover-procedure/ or double-blind procedure/ or randomized controlled trial/ or single-blind procedure/
10. 8 and 9
11. limit 10 to exclude medline journal

***C. MEDLINE via Ovid SP***

1. exp Heart Failure/ or (((Heart or Cardiac or Cardial or Myocardial or Coronary) adj2 (Failure? Or Decompensation? or Incompetence? Or Insufficien$ or "stand still" or standstill)) or Cardio Renal Syndrome? or Cardiorenal Syndrome? or Cardio-Renal Syndrome? or Reno cardiac Syndrome? or Renocardiac Syndrome? or Reno-cardiac Syndrome? or Paroxysmal Dyspnea? or Cardiac Asthma or Cardiac Edema? Or heart edema? Or ((cardiogenic or cardiac or cardiovascular or heart) adj shock$) or ((cardiopulmonary or cardiorespiratory) adj (insufficiency or arrest$ or collaps$ or failure?)) or CHF or left ventricular diastolic dysfunction or forward failure? Or low cardiac output? Or low heart output? Or low output syndrome? or ((cardiac or circulation or circulatory or heart or cardiopulmonary) adj arrest?) or asystole or asystolia or asystoly or heart arrhythmia? Or cardiac death? or heart death? or heart outflow tract obstruction? or ventricular outflow tract obstruction? or ventricular outflow obstruction? or ventricle obstruction? Or heart outflow obstruction? Or heart obstruction? Or ventric$ failure? Or ventric$ insufficien$ or ventric$ dysfunction? Or systolic dysfunction? Or diastolic overload? or systolic overload? or heart overload? or ventric$ overload? Or ventric$ tension overload? Or ventric$ pressure overload? Or ventric$ volume overload? Or ventric$ strain? Or high cardiac output? or high output failure? or propofol infusion syndrome? or propofol related infusion syndrome?).mp.
2. Fosinopril/ or Lisinopril/ or Perindopril/ or exp Enalapril/ or Captopril/ or (Accupril or Accuprin or Accupro or Accupron or Ace-bloc or Acenorm or Acenor-m or Aceon or Acepress or Acepril or Aceprilex or Acequin or Acerbon or Aceril or Aceten or Acuitel or Acuprel or Acupril or Adocor or Alapril or Alfaken or Alopresin or Altran or Amprace or Apuzin or Asig or Asisten or Benazapril or Benazepril or Benzazepril or Bpnorm or Briem or Capace or Capocard or Caposan or Capoten or Capotena or Capotril or Capril or Captace or Captensin or Capti or Captoflux or Captohexal or Captolane or Captomax or Capton or Captopren or Captopril or Captoprilan or Captoril or Captral or Carace or Cardiopril or Cardipril or Catona or Catoplin or Catopril or Cesplon or "CGS-14824A" or "CGS-14824-A" or "CI 906" or "CI906" or "CI-906" or Cibacen or Cibacene or Cipril or Conan or Coric or Coversum or Coversyl or Cryopril or Dapril or Debax or Dexacap or Dynacil or Ecapres or Ecaten or Ednyt or Enalapril$ or Epicordin or Epsitron or Farcopril or Farmoten or Fibsol or Fosenopril or Fosinil or Fosinonorm or Fosinopril or Fosinorm or Fosipres or Fositen or Fositens or Fovas or Fozitec or Hiperil or Hiperlex or Hypopress or Hypotensor or "Ici 209000" or "Ici pharma 209000" or "Ici209000" or Inopril or Insucar or Iopril or Isopresol or Katopil or Ketanine or Keyerpril or Korec or Labopal or Lapril or Linopril or Linvas or Lipril or "Lisi abz" or Lisibeta or Lisigamma or Lisihexal or Lisinopril or Lisipril or Lisodur or Lisopress or Lisopril or Lisoril or Lispril or Listril or Locap or Lopirin or Lopril or Lotensin or Lysinopril or Medepres or Midrat or Minitent or "Mk 0521" or "MK 421" or "MK 422" or "Mk 521" or "Mk 522" or "Mk0521" or "MK421" or "MK-421" or "MK422" or "MK-422" or "Mk521" or "MK-521" or "Mk522" or Monopril or Naprilene or Newace or Nolectin or Noperten or Novatec or "Oltens ge" or "Pd 109452 2" or "Pd 1094522" or "PD 109452-2" or "PD-109,452-2" or "Pd109452 2" or "Pd1094522" or Perindopril or Perstarium or Petacilon or Pirindopril or Praten or "Pres iv" or Presiten or Prestarium or Primace or Prinil or Prinivil or Quinalapril or Quinapril or Quinaten or Quinazil or Quinipril or Renitec or Renitek or Rilcapton or Ropril or "S 9490" or "S 9490 3" or "S 94903" or "S 9490-3" or "S9490" or "S-9490" or Sapril or Sinopril or Smarten or "SQ 14,225" or "SQ 14,534" or "SQ 14225" or "SQ 14534" or "Sq 28 555" or "SQ 28,555" or "Sq 28555" or "SQ 28555" or "SQ14,225" or "SQ-14,225" or "SQ14,534" or "SQ-14,534" or "SQ14225" or "SQ-14225" or "SQ14534" or "SQ-14534" or "SQ28,555" or "SQ-28,555" or "Sq28.555" or "SQ28555" or "SQ-28555" or Staril or Tenofax or Tensicap or Tensiomen or Tensiomin or "Tenso Stop" or Tensobon or Tensocardil or Tensoprel or Tensopril or Tensoril or Tensyn or Tenzib or Topace or Toprilem or Typril-ace or Vasopril or Vasosta or Vasotec or Vivatec or Xanef or Zapto or Zestomax or Zestril or Zorkaptil).mp.
3. (randomized controlled trial or controlled clinical trial or pragmatic clinical trial).pt. or (randomi?ed or randomly).ab. or placebo.ab. or drug therapy.fs. or trial.ab. or groups.ab. not (animals not (humans and animals)).sh.
4. 1 and 2 and 3

***D. PubMed***

(((heart[All Fields] OR cardiac[All Fields] OR cardial[All Fields] OR myocardial[All Fields] OR coronary[All Fields]) AND (failure*[All Fields] OR decompensation*[All Fields] OR incompetence*[All Fields] OR insufficien*[All Fields] OR "stand still"[All Fields] OR standstill[All Fields])) OR "cardio renal syndrome"[All Fields] OR "cardiorenal syndrome"[All Fields] OR "cardio-renal syndrome"[All Fields] OR "reno cardiac syndrome"[All Fields] OR "renocardiac syndrome"[All Fields] OR "reno-cardiac syndrome"[All Fields] OR "paroxysmal dyspnea"[All Fields] OR "cardiac asthma"[All Fields] OR "cardiac edema"[All Fields] OR "heart edema"[All Fields] OR ((cardiogenic[All Fields] OR cardiac[All Fields] OR cardiovascular[All Fields] OR heart[All Fields]) AND shock*[All Fields]) OR ((cardiopulmonary[All Fields] OR cardiorespiratory[All Fields]) AND (insufficiency[All Fields] OR arrest*[All Fields] OR collaps*[All Fields] OR failure*[All Fields])) OR chf[All Fields] OR "left ventricular diastolic dysfunction"[All Fields] OR "forward failure"[All Fields] OR "low cardiac output"[All Fields] OR "low heart output"[All Fields] OR "low output syndrome"[All Fields] OR ((cardiac[All Fields] OR circulation[All Fields] OR circulatory[All Fields] OR heart[All Fields] OR cardiopulmonary[All Fields]) AND arrest*[All Fields]) OR asystole[All Fields] OR asystolia[All Fields] OR asystoly[All Fields] OR "heart arrhythmia"[All Fields] OR "cardiac death"[All Fields] OR "heart death"[All Fields] OR "heart outflow tract obstruction"[All Fields] OR "ventricular outflow tract obstruction"[All Fields] OR "ventricular outflow obstruction"[All Fields] OR "ventricle obstruction"[All Fields] OR "heart outflow obstruction"[All Fields] OR "heart obstruction"[All Fields] OR "ventricular failure"[All Fields] OR "ventricular insufficien"[All Fields] OR "ventricular dysfunction"[All Fields] OR "systolic dysfunction"[All Fields] OR "diastolic overload"[All Fields] OR "systolic overload"[All Fields] OR "heart overload"[All Fields] OR "ventricular overload"[All Fields] OR "ventricular tension overload"[All Fields] OR "ventricular pressure overload"[All Fields] OR "ventricular volume overload"[All Fields] OR "ventricular strain"[All Fields] OR "high cardiac output"[All Fields] OR "high output failure"[All Fields] OR "propofol infusion syndrome"[All Fields] OR "propofol related infusion syndrome"[All Fields] OR "heart failure"[mesh]) AND ("fosinopril"[mesh] OR "lisinopril"[mesh] OR "perindopril"[mesh] OR "enalapril"[mesh] OR "captopril"[mesh] OR accupril[All Fields] OR accuprin[All Fields] OR accupro[All Fields] OR accupron[All Fields] OR ace-bloc[All Fields] OR acenorm[All Fields] OR acenor-m[All Fields] OR aceon[All Fields] OR acepress[All Fields] OR acepril[All Fields] OR aceprilex[All Fields] OR acequin[All Fields] OR acerbon[All Fields] OR aceril[All Fields] OR aceten[All Fields] OR acuitel[All Fields] OR acuprel[All Fields] OR acupril[All Fields] OR adocor[All Fields] OR alapril[All Fields] OR alfaken[All Fields] OR alopresin[All Fields] OR altran[All Fields] OR amprace[All Fields] OR apuzin[All Fields] OR asig[All Fields] OR asisten[All Fields] OR benazapril[All Fields] OR benazepril[All Fields] OR benzazepril[All Fields] OR bpnorm[All Fields] OR briem[All Fields] OR capace[All Fields] OR capocard[All Fields] OR caposan[All Fields] OR capoten[All Fields] OR capotena[All Fields] OR capotril[All Fields] OR capril[All Fields] OR captace[All Fields] OR captensin[All Fields] OR capti[All Fields] OR captoflux[All Fields] OR captohexal[All Fields] OR captolane[All Fields] OR captomax[All Fields] OR capton[All Fields] OR captopren[All Fields] OR captopril[All Fields] OR captoprilan[All Fields] OR captoril[All Fields] OR captral[All Fields] OR carace[All Fields] OR cardiopril[All Fields] OR cardipril[All Fields] OR catona[All Fields] OR catoplin[All Fields] OR catopril[All Fields] OR cesplon[All Fields] OR "cgs-14824a"[All Fields] OR "cgs-14824-a"[All Fields] OR "ci 906"[All Fields] OR "ci906"[All Fields] OR "ci-906"[All Fields] OR cibacen[All Fields] OR cibacene[All Fields] OR cipril[All Fields] OR conan[All Fields] OR coric[All Fields] OR coversum[All Fields] OR coversyl[All Fields] OR cryopril[All Fields] OR dapril[All Fields] OR debax[All Fields] OR dexacap[All Fields] OR dynacil[All Fields] OR ecapres[All Fields] OR ecaten[All Fields] OR ednyt[All Fields] OR enalapril*[All Fields] OR epicordin[All Fields] OR epsitron[All Fields] OR farcopril[All Fields] OR farmoten[All Fields] OR fibsol[All Fields] OR fosenopril[All Fields] OR fosinil[All Fields] OR fosinonorm[All Fields] OR fosinopril[All Fields] OR fosinorm[All Fields] OR fosipres[All Fields] OR fositen[All Fields] OR fositens[All Fields] OR fovas[All Fields] OR fozitec[All Fields] OR hiperil[All Fields] OR hiperlex[All Fields] OR hypopress[All Fields] OR hypotensor[All Fields] OR "ici 209000"[All Fields] OR "ici pharma 209000"[All Fields] OR "ici209000"[All Fields] OR inopril[All Fields] OR insucar[All Fields] OR iopril[All Fields] OR isopresol[All Fields] OR katopil[All Fields] OR ketanine[All Fields] OR keyerpril[All Fields] OR korec[All Fields] OR labopal[All Fields] OR lapril[All Fields] OR linopril[All Fields] OR linvas[All Fields] OR lipril[All Fields] OR "lisi abz"[All Fields] OR lisibeta[All Fields] OR lisigamma[All Fields] OR lisihexal[All Fields] OR lisinopril[All Fields] OR lisipril[All Fields] OR lisodur[All Fields] OR lisopress[All Fields] OR lisopril[All Fields] OR lisoril[All Fields] OR lispril[All Fields] OR listril[All Fields] OR locap[All Fields] OR lopirin[All Fields] OR lopril[All Fields] OR lotensin[All Fields] OR lysinopril[All Fields] OR medepres[All Fields] OR midrat[All Fields] OR minitent[All Fields] OR "mk 0521"[All Fields] OR "mk 421"[All Fields] OR "mk 422"[All Fields] OR "mk 521"[All Fields] OR "mk 522"[All Fields] OR "mk0521"[All Fields] OR "mk421"[All Fields] OR "mk-421"[All Fields] OR "mk422"[All Fields] OR "mk-422"[All Fields] OR "mk521"[All Fields] OR "mk-521"[All Fields] OR "mk522"[All Fields] OR monopril[All Fields] OR naprilene[All Fields] OR newace[All Fields] OR nolectin[All Fields] OR noperten[All Fields] OR novatec[All Fields] OR "oltens ge"[All Fields] OR "pd 109452 2"[All Fields] OR "pd 1094522"[All Fields] OR "pd 109452-2"[All Fields] OR "pd-109,452-2"[All Fields] OR "pd109452 2"[All Fields] OR "pd1094522"[All Fields] OR perindopril[All Fields] OR perstarium[All Fields] OR petacilon[All Fields] OR pirindopril[All Fields] OR praten[All Fields] OR "pres iv"[All Fields] OR presiten[All Fields] OR prestarium[All Fields] OR primace[All Fields] OR prinil[All Fields] OR prinivil[All Fields] OR quinalapril[All Fields] OR quinapril[All Fields] OR quinaten[All Fields] OR quinazil[All Fields] OR quinipril[All Fields] OR renitec[All Fields] OR renitek[All Fields] OR rilcapton[All Fields] OR ropril[All Fields] OR "s 9490"[All Fields] OR "s 9490 3"[All Fields] OR "s 94903"[All Fields] OR "s 9490-3"[All Fields] OR "s9490"[All Fields] OR "s-9490"[All Fields] OR sapril[All Fields] OR sinopril[All Fields] OR smarten[All Fields] OR "sq 14,225"[All Fields] OR "sq 14,534"[All Fields] OR "sq 14225"[All Fields] OR "sq 14534"[All Fields] OR "sq 28 555"[All Fields] OR "sq 28,555"[All Fields] OR "sq 28555"[All Fields] OR "sq 28555"[All Fields] OR "sq14,225"[All Fields] OR "sq-14,225"[All Fields] OR "sq14,534"[All Fields] OR "sq-14,534"[All Fields] OR "sq14225"[All Fields] OR "sq-14225"[All Fields] OR "sq14534"[All Fields] OR "sq-14534"[All Fields] OR "sq28,555"[All Fields] OR "sq-28,555"[All Fields] OR "sq28.555"[All Fields] OR "sq28555"[All Fields] OR "sq-28555"[All Fields] OR staril[All Fields] OR tenofax[All Fields] OR tensicap[All Fields] OR tensiomen[All Fields] OR tensiomin[All Fields] OR "tenso stop"[All Fields] OR tensobon[All Fields] OR tensocardil[All Fields] OR tensoprel[All Fields] OR tensopril[All Fields] OR tensoril[All Fields] OR tensyn[All Fields] OR tenzib[All Fields] OR topace[All Fields] OR toprilem[All Fields] OR typril-ace[All Fields] OR vasopril[All Fields] OR vasosta[All Fields] OR vasotec[All Fields] OR vivatec[All Fields] OR xanef[All Fields] OR zapto[All Fields] OR zestomax[All Fields] OR zestril[All Fields] OR zorkaptil[All Fields]) AND (randomized controlled trial[pt] OR controlled clinical trial[pt] OR pragmatic clinical trial[pt] OR randomized[tiab] OR randomly[tiab] OR placebo[tiab] OR randomly[tiab] OR trial[tiab] OR groups[tiab]) NOT MEDLINE[sb]

Supplemental Table 1. The pairwise and network results for all cause-mortality.

| Captopril | 0.78(0.23,1.76) | 0.37(0.04,1.07) | 9.25(0.76,31.02) | 1.98(0.58,4.66) |
| --- | --- | --- | --- | --- |
| 0.68(0.23,2.03) | Enalapril | 0.68(0.05,2.23) | 16.58(0.97,57.6) | 3.47(0.68,9.58) |
| 0.42(0.08,2.19) | NA | Lisinopril | **65.6(1.91,239.6)** | **14.65(1.23,49.5)** |
| NA | NA | NA | Ramipril | 0.53(0.07,1.51) |
| 1.41(0.44,4.52) | 1.79(0.18,18.6) | 7.39(0.46,118.1) | 0.41(0.11,1.44) | Placebo |

For all cause-mortality, odds ratios (ORs) lower than 1 favored the column-defining treatment. Pairwise comparisons were shown in the bottom left. Network comparisons were shown in the upper right. The number which was painted by a style of overstriking indicated there was a significant difference between two treatments.

Significant results: network meta-analyses indicated that lisinopril groups caused higher all-cause mortality compared with ramipril groups or placebo groups.

Supplemental Table 2. The pairwise and network results for stroke volume.

| Captopril | -0.02(-0.69,0.64) | 0.09(-0.86,1.05) | 0.42(-0.35,1.24) |
| --- | --- | --- | --- |
| -0.22(-0.73,0.3) | Enalapril | 0.11(-1.04,1.28) | 0.44(-0.21,1.14) |
| 0.09(-0.16,0.34) | NA | Lininopril | 0.33(-0.87,1.59) |
| **1(0.11,1.87)** | 0.26(-0.77,1.29) | NA | Placebo |

For stroke volume, standard mean differences (SMDs) lower than 0 favored the column-defining treatment. Pairwise comparisons were shown in the bottom left. Network comparisons were shown in the upper right. The number which was painted by a style of overstriking indicated there was a significant difference between two treatments.

Significant results: pairwise meta-analyses indicated that captopril groups significantly improved stroke volume compared with placebo groups.

Supplemental Table 3. The pairwise and network results for ejection fraction.

| Captopril | -0.14(-1.2,0.87) | 0(-0.83,0.83) | 0.13(-0.74,0.99) |
| --- | --- | --- | --- |
| NA | Enalapril | 0.14(-1.17,1.52) | 0.27(-0.3,0.87) |
| 0(-0.25,0.25) | NA | Lisinopril | 0.13(-1.08,1.32) |
| 0.14(-0.23,0.51) | 0.25(-0.31,0.8) | NA | Placebo |

For ejection fraction, standard mean differences (SMDs) lower than 0 favored the column-defining treatment. Pairwise comparisons were shown in the bottom left. Network comparisons were shown in the upper right. The number which was painted by a style of overstriking indicated there was a significant difference between two treatments.

Significant results: none.

Supplemental Table 4. The pairwise and network results for systolic blood pressure.

| Captopril | 0.36(-0.42, 1.17) | -0.29(-0.94,0.34) | 0.79(-0.19,1.8) | -0.24(-0.91,0.43) |
| --- | --- | --- | --- | --- |
| NA | Enalapril | -0.65(-1.7,0.35) | 0.43(-0.16,1.02) | **-0.6(-1.03,-0.18)** |
| **-0.3(-0.55,-0.05**) | NA | Lisinopril | 1.08(-0.08,2.28) | 0.05(-0.88,0.99) |
| NA | 0.44(-0.07,0.95) | NA | Trandolapril | **-1.03(-1.77,-0.29)** |
| -0.24(-0.59,0.11) | **-0.59(-1.05,-0.13)** | NA | NA | Placebo |

For systolic blood pressure, standard mean differences (SMDs) lower than 0 favored the column-defining treatment. Pairwise comparisons were shown in the bottom left. Network comparisons were shown in the upper right. The number which was painted by a style of overstriking indicated there was a significant difference between two treatments.

Significant results: pairwise meta-analyses indicated captopril groups significantly reduced systolic blood pressure compared with lisinopril groups and enalapril significantly reduced systolic blood pressure in contrast to placebo groups. Network meta-analyses showed enalapril and trandolapril groups significantly reduced systolic blood pressure compared to placebo groups.

Supplemental Table 5. The pairwise and network results for diastolic blood pressure.

| Captopril | 0.4(-0.48,1.28) | -0.18(-0.82,0.47) | 0.56(-0.49,1.62) | -0.01(-0.68,0.64) |
| --- | --- | --- | --- | --- |
| NA | Enalapril | -0.58(-1.65,0.50) | 0.16(-0.43,0.75) | -0.41(-0.99,0.16) |
| -0.18(-0.43,0.07) | NA | Lisinopril | 0.74(-0.48,1.95) | 0.16(-0.76,1.08) |
| NA | 0.17(-0.34,0.67) | NA | Trandopril | -0.41(-0.99,0.16) |
| -0.01(-0.35,0.33) | -0.41(-0.83,0.01) | NA | NA | Placebo |

For diastolic blood pressure, standard mean differences (SMDs) lower than 0 favored the column-defining treatment. Pairwise comparisons were shown in the bottom left. Network comparisons were shown in the upper right. The number which was painted by a style of overstriking indicated there was a significant difference between two treatments.

Significant results: none.

Supplemental Table 6. The pairwise and network results for mean arterial pressure.

| Captopril | 0.19(-0.11,0.48) | -0.28(-0.63,0.05) |
| --- | --- | --- |
| 0.12(-0.25,0.49) | Enalapril | -0.47(-0.85,-0.1) |
| -0.15(-0.45,0.16) | -0.71(-1.31,-0.11) | Placebo |

For mean arterial pressure, standard mean differences (SMDs) lower than 0 favored the column-defining treatment. Pairwise comparisons were shown in the bottom left. Network comparisons were shown in the upper right. The number which was painted by a style of overstriking indicated there was a significant difference between two treatments.

Significant results: none

Supplemental Table 7. The pairwise and network results for cough.

| Captopril | 0.64(0.1,1.78) | **76.2(1.56,149.3)** |
| --- | --- | --- |
| 0.54(0.14,2.15) | Enalapril | **274.4(2.4,512.9)** |
| 6.44(0.76,54.82) | 7.6(0.15,383.3) | Placebo |

For cough, odds ratios (ORs) lower than 1 favored the column-defining treatment. Pairwise comparisons were shown in the bottom left. Network comparisons were shown in the upper right. The number which was painted by a style of overstriking indicated there was a significant difference between two treatments.

Significant results: network meta-analyses indicated captopril and enalapril groups lead to higher incidence of cough compared with placebo groups.

Supplemental Table 8. The pairwise and network results for deterioration of renal function.

| Captopril | **0.04(0.002,0.14)** | 0.53(0.001,2.21) | NA |
| --- | --- | --- | --- |
| 0.06(0.003,1.3) | Enalapril | 116.8(0.05,256) | NA |
| 0.14(0.009,2.25) | NA | Lininopril | NA |
| NA | NA | 7.5(0.15,377.9) | Placebo |

For deterioration of renal function, odds ratios (ORs) lower than 1 favored the column-defining treatment. Pairwise comparisons were shown in the bottom left. Network comparisons were shown in the upper right. The number which was painted by a style of overstriking indicated there was a significant difference between two treatments.

Significant results: network meat-analyses indicated enalapril groups caused higher incidence of deterioration of renal function compared with captopril groups.

Supplemental Table 9. The pairwise and network results for gastrointestinal discomfort.

| Captopril | 0.68(0.15,1.72) | 5.42(0.19,17.7) | 7.88(0.46,26.74) |
| --- | --- | --- | --- |
| 0.37(0.01,10) | Enalapril | 10.01(0.41,33.14) | 17.9(0.74,61.28) |
| NA | 1.99(0.18,22.15) | Lisinopril | 10.48(0.09,36.84) |
| **7.7(1.06,55.76)** | 0.17(0.003,7.44) | NA | Placebo |

For gastrointestinal discomfort, odds ratios (ORs) lower than 1 favored the column-defining treatment. Pairwise comparisons were shown in the bottom left. Network comparisons were shown in the upper right. The number which was painted by a style of overstriking indicated there was a significant difference between two treatments.

Significant results: pairwise meat-analyses indicated captopril groups caused higher incidence of gastrointestinal discomfort compared with placebo groups.
